# Conditional Deletion of Translin/Trax in Dopaminergic Neurons Reveals No Impact on Psychostimulant Behaviors or Adiposity

**DOI:** 10.3390/biom15071040

**Published:** 2025-07-17

**Authors:** Yunlong Liu, Renkun Wu, Gaiyuan Geng, Helian Yang, Chunmiao Wang, Mengtian Ren, Xiuping Fu

**Affiliations:** School of Life Sciences and School of Chemistry, Tiangong University, Tianjin 300387, China; liuyunlong@tiangong.edu.cn (Y.L.); 2330131352@tiangong.edu.cn (R.W.); 2431131389@tiangong.edu.cn (G.G.); 2431131415@tiangong.edu.cn (H.Y.); 2431161548@tiangong.edu.cn (C.W.); mengtianren@tiangong.edu.cn (M.R.)

**Keywords:** cocaine, amphetamine, Translin/Trax, dopaminergic neurons, microRNAs

## Abstract

Despite the abundant expression of the microRNA-degrading Translin (TN)/Trax (TX) complex in midbrain dopaminergic (DA) neurons and its implication in neuropsychiatric disorders, its cell-autonomous roles in metabolic and behavioral responses remain unclear. To address this, we generated DA neuron-specific conditional knockout (cKO) mice for *Tsn* (TN) or *Tsnax* (TX) using DAT-Cre. Immunostaining confirmed efficient TX loss in *Tsnax* cKO DA neurons without affecting TN, while *Tsn* deletion abolished TX expression, revealing asymmetric protein dependency. Body composition analysis showed no alterations in adiposity in either cKO model. Locomotor responses to acute or repeated administration of cocaine (20 mg/kg) or amphetamine (2.5 mg/kg) were unchanged in *Tsn* or *Tsnax* cKO mice. Furthermore, amphetamine-induced conditioned place preference (1 mg/kg) was unaffected. These results demonstrate that the TN/TX complex within DA neurons is dispensable for regulating adiposity, psychostimulant-induced locomotion (both acute and sensitized), or amphetamine reward-related behavior, suggesting its critical functions may lie outside these specific domains.

## 1. Introduction

In the mammalian central nervous system, midbrain dopaminergic (DA) neurons serve as the principal source of dopamine, a critical neurotransmitter involved in regulating motor coordination, emotional processing, memory, and neuroendocrine function [1]. Dysregulation of the dopaminergic system has been implicated in a wide range of neurological and psychiatric disorders, including Parkinson’s disease (PD), addiction, schizophrenia, attention deficit hyperactivity disorder (ADHD), and depression [2]. Therefore, elucidating the regulatory mechanisms governing dopamine signaling is essential for the development of targeted therapeutic strategies aimed at restoring physiological function and behavioral homeostasis in affected individuals.

MicroRNAs (miRNAs), a class of small non-coding RNAs comprising 19–25 nucleotides, function as potent post-transcriptional regulators of gene expression [3,4]. They exert their effects by binding to specific sequences, known as miRNA recognition elements (MREs), typically located within the 3′-untranslated regions (UTRs) of target mRNAs, thereby repressing translation or promoting mRNA degradation [5,6]. This regulatory mechanism is critical for modulating neuroplasticity, synaptic consolidation, and gene expression within the central nervous system (CNS) [7,8]. In midbrain DA neurons, miRNAs are integral to the regulation of neuronal development, maintenance, and function, supporting key physiological roles such as motor control and motivational behavior [1]. The conditional ablation of Dicer—a central enzyme in miRNA biogenesis—in mouse models has highlighted the essential role of miRNAs in CNS development, DA neuron differentiation, and survival. The loss of Dicer leads to midbrain abnormalities, DA neuronal degeneration, and apoptosis, closely recapitulating pathological features of Parkinson’s disease (PD) [9,10,11]. Given the broad impact of the dopaminergic system on neuropsychiatric conditions, recent advances uncovering the regulatory functions of miRNAs in modulating DA neuron activity have generated significant interest in their potential to influence dopamine-mediated processes and the pharmacological actions of psychostimulants.

To elucidate the physiological roles of microRNA signaling in the dopamine system, we have initiated a study on the Translin/Trax (TN/TX) complex, a heteromeric RNase that specifically degrades a small subset of microRNAs [12,13]. Upon stimulation, TN/TX promotes rapid microRNA decay, lifting repression on target mRNAs and enabling de novo protein synthesis. This activity positions TN/TX as a key regulator of translation-dependent cellular plasticity. In our previous study, we demonstrated that TN and TX are expressed in both midbrain dopaminergic and striatal neurons [14], which are critically involved in the effects of psychostimulants [15,16,17]. Moreover, the microRNA system has been shown to significantly contribute to the regulation of behavioral responses elicited by these drugs [18,,19,20,21], as evidenced by findings that the deletion of Ago2 in D2 receptor-expressing striatal neurons suppresses cocaine-induced place preference and self-administration []. Also, miR-495 overexpression in the nucleus accumbens reduces cocaine-seeking without affecting food reinforcement [19]. Thus, we examined the impact of translin deletion on the locomotor response to amphetamine, a classic behavioral assay mediated by the mesolimbic dopamine pathway [22]. Initial studies using global Translin (*Tsn*) knockout (KO) mice revealed a significant elevation in adiposity. This metabolic phenotype confounded behavioral interpretations, as it led to increased brain levels of amphetamine and, consequently, to an enhanced locomotor response [14]. To circumvent this confounding factor, we utilized conditional *Tsn* knockout (KO) mice in which global gene deletion was induced during adulthood, a model that maintains normal adiposity. In this system, we observed that the locomotor response to a single dose of amphetamine remained unaltered [14,23]. Additionally, we assessed the locomotor effects of cocaine—another psychostimulant commonly used to probe dopaminergic function—and found that while the acute response was preserved, the locomotor response to repeated cocaine exposure was significantly impaired in *Tsn* KO mice [24]. Moreover, the TN/TX RNase complex has been implicated in the regulation of various forms of enduring synaptic plasticity [25]. Collectively, these findings have sparked growing interest in elucidating the role of the TN/TX microRNA-degrading enzyme in modulating the dopamine system.

To investigate the function of the TN/TX RNase complex in DA neurons with respect to metabolic and behavioral outcomes, we examined whether the conditional knockout of *Tsn* or *Tsnax* in DA neurons would impact body fat percentage or locomotor responses to amphetamine or cocaine. Our results demonstrate that the conditional knockout of *Tsn* or *Tsnax* in DA neurons does not alter body fat composition. Furthermore, neither acute nor repeated administration of amphetamine or cocaine elicited changes in locomotor activity as a result of these deletions. Similarly, amphetamine-induced conditioned place preference (CPP) was unaffected by the conditional loss of either gene in DA neurons. Collectively, these findings suggest that although the TN/TX complex is abundantly expressed in DA neurons, it may not play a critical role in regulating adiposity or psychostimulant-induced behavioral responses, including locomotor activity and reward-related behavior as assessed by CPP.

## 2. Materials and Methods

### 2.1. Animals

All mice were housed in ventilated racks and maintained on a 12 h/12 h light/dark cycle with access to food and water ad libitum. All experimental procedures and animal protocols were approved by the Animal Welfare and Ethical Review Committee of Tiangong University for animal research (protocol code: 2024-002; date of approval: 14 March 2024) and was conducted by following the animal research guidelines of the Tiangong University. Both male and female mice were used for body composition assays, as indicated. Only male mice were used in all other assays.

Conditional alleles of *Tsn* or *Tsnax* were engineered on the C57BL/6J genetic background via CRISPR/Cas9-mediated genome editing, as previously detailed in Fu et al. [23]. The DAT-Cre line was obtained from JAX labs (#006660). For all experiments, 7–8 mice per group were used, with a minimum of 7 animals in each group to ensure statistical reliability.

### 2.2. Immunostaining

Three- to four-month-old male mice were deeply anesthetized with chloral hydrate (400 mg/kg, i.p.) and subjected to transcardial perfusion using 4% paraformaldehyde in 0.1 M phosphate-buffered saline (PBS; pH 7.4). The brains were removed, post-fixed in 4% paraformaldehyde for a minimum of 24 h, rinsed in PBS, and cryoprotected in 30% sucrose until fully submerged. Coronal brain sections (30 μm) were subsequently prepared using a freezing microtome.

For TN immunostaining, heat-induced antigen retrieval was performed by incubating the sections in 10 mM sodium citrate at 70 °C for 30 min. Following cooling to room temperature, the sections were rinsed with PBS, blocked with 3% BSA and 0.1% Triton X-100 for 1 h, and incubated overnight at 4 °C with primary antibodies targeting TN [26] and tyrosine hydroxylase (TH; MilliporeSigma, Burlington, MA, USA). The next day, sections were treated with Alexa Fluor 488-conjugated goat anti-rabbit and Alexa Fluor 594-conjugated goat anti-mouse secondary antibodies (Jackson ImmunoResearch Laboratories, West Grove, PA, USA) in 1.5% normal goat serum (Vector Laboratories, Burlingame, CA, USA) for 1 h at room temperature. After final rinses, the sections were mounted and coverslipped.

For TX immunostaining, sections underwent a similar pre-treatment process. After blocking, they were incubated overnight at 4 °C with primary antibodies against TX [27] and TH. This was followed by sequential incubations with biotinylated goat anti-rabbit and Alexa Fluor 594 goat anti-mouse antibodies, both diluted in 1.5% normal goat serum. Amplification was achieved using the ABC method (Vector Laboratories, Burlingame, CA, USA) for 90 min, followed by TSA tyramide signal amplification (PerkinElmer, Waltham, MA, USA) for 10 min. After thorough washing, sections were mounted on slides and coverslipped.

All images were obtained with a Zeiss LSM 800 confocal microscope (Carl Zeiss AG, Oberkochen, Germany).

### 2.3. Open-Field Locomotor Activity

Changes in locomotor activity in response to amphetamine or cocaine were assessed in an open field arena (50 × 50 cm). A 3- to 4-month old male mice were first allowed to habituate to the arena for 30 min. Following habituation to the context, mice were given an i.p. injection of saline and then placed back in the center of the open field and allowed to explore the arena for another 30 min. At this point, mice were given an intraperitoneal (i.p.) injection of D-amphetamine hemisulfate (2.5 mg/kg of total body weight; MilliporeSigma) or cocaine (20 mg/kg of total body weight; MilliporeSigma), placed back in the arena and then monitored for an additional 50 min.

### 2.4. Conditioned Place Preference (CPP)

The chamber for CPP was designed with three interconnected compartments: two lateral compartments A/B (20 cm × 30 cm for each) and a middle compartment (10 cm × 30 cm). Compartment A was packed with fine padding, compartment B packed with coarse padding, and the middle compartment did not have any floor covering. Briefly, the first day mice underwent habituation during which they were allowed to freely explore all compartments without injection. On the second day mice underwent a pre-conditioning test, in which mice were i.p. injected with saline, placed in the middle compartment, and allowed to explore all the compartments. Overall, there was no preference for one compartment versus the other during this pre-test, indicating that the apparatus was unbiased. Mice were then i.p. injected with saline and randomly confined to one compartment and, on alternating days, were injected with amphetamine (1 mg/kg) and confined to the alternate compartment for a total of 3 pairings each. The day following the last conditioning session, the mice were injected with saline and allowed free access to all compartments. The time spent in the compartment in which they received amphetamine injections was used to index the direction and magnitude of the place-conditioning.

### 2.5. Body Composition

Nuclear magnetic resonance (NMR) scanning (EchoMRI-100, EchoMRI LLC, Houston, TX, USA) was employed to assess body composition, while body length was recorded as the distance between the snout tip and tail base. In the measurement of subcutaneous and visceral fat depots, unanesthetized mice were placed in a restraint tube and inserted into the NMR. We validated the NMR results by ether extraction at the end of the experiments.

### 2.6. Statistical Analysis

All data are expressed as mean ± SEM. Statistical analyses were performed using GraphPad Prism 8 (GraphPad Software, La Jolla, CA, USA). For comparisons involving a single variable, Student’s *t*-test was applied. In experiments with repeated measures and multiple factors, two-way RM ANOVA was conducted, followed by Bonferroni post hoc tests for pairwise comparisons. A *p*-value less than 0.05 was considered statistically significant.

## 3. Results

### 3.1. Conditional Deletion of Tsnax from DA Neurons

**Figure 1:**
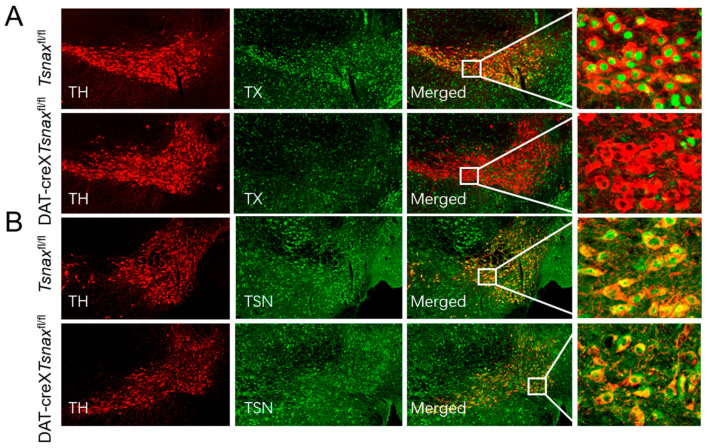
Conditional knockout of *Tsnax* from DA neurons. Dual immunofluorescence staining of midbrain sections for TH (red) alongside TX or TN (green) demonstrates that in DAT-cre×*Tsnax*^fl/fl^ mice (**A**) TX expression was totally absent but (**B**) TN expression was normal. Scale bar, 30 μm.

Our prior study demonstrated that TN and TX are co-expressed in midbrain DA neurons [14]. To investigate their cell-autonomous functions, we generated DA neuron-specific *Tsn* knockout mice (DAT-cre×*Tsn*^fl/fl^) confirmed that locomotor responses to acute amphetamine administration were unaltered [14]. Since TX could act independently of translin, for example, binding to and activating ATM kinase [28], we generated mice homozygous for the floxed *Tsnax* allele (*Tsnax*^fl/fl^) and hemizygous for the DAT-Cre allele. Immunofluorescence analysis for TX and tyrosine hydroxylase (TH) demonstrated a complete loss of TX expression in midbrain dopaminergic neurons (Figure 1A). Notably, although *Tsn* deletion leads to the absence of TX expression [14], *Tsnax* deletion does not affect TN protein levels (Figure 1B), suggesting that TN may have distinct and independent functions in physiological processes.

### 3.2. Effect of Conditional Deletion of Tsn or Tsnax from DA Neurons on Adiposity

**Figure 2:**
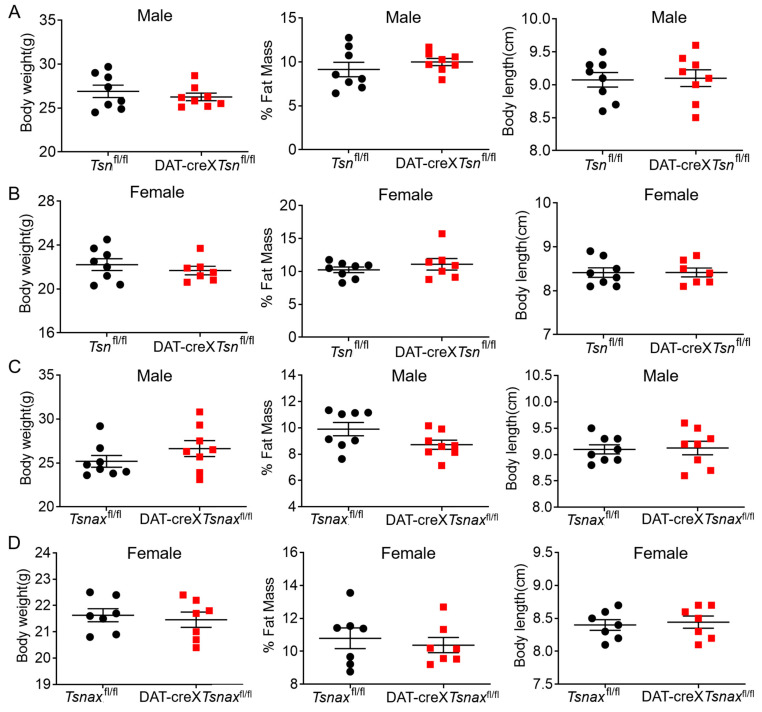
Conditional deletion of *Tsn* or *Tsnax* from DA neurons does not affect adiposity. Both male (**A**) and female (**B**) DAT-cre×*Tsn*^fl/fl^ mice exhibit body weight, fat mass, and body length comparable to those of *Tsn*^fl/fl^ controls. Similarly, conditional deletion of *Tsnax* in dopaminergic neurons does not alter body weight, fat mass, or body length in either male (**C**) or female (**D**) mice. Each group included 7–8 animals. Data are presented as mean ± SEM, and statistical significance was assessed using Student’s *t*-test.

Given that global *Tsn* knockout (KO) mice exhibit significantly increased adiposity, which influences amphetamine pharmacokinetics [14], and that midbrain DA neurons play a key role in regulating metabolic homeostasis [29,30,31], we first examined whether the conditional knockout of *Tsn* or *Tsnax* in midbrain DA neurons would recapitulate the adiposity phenotype observed in global *Tsn* KO mice. Body composition analysis revealed that there were negligible differences in body weight, percentage of fat mass, and body length between *Tsn*^fl/fl^ and DAT-cre×*Tsn*^fl/fl^ mice (Figure 2A,B). Similar results were shown between *Tsnax*^fl/fl^ and DAT-cre×*Tsnax*^fl/fl^ mice (Figure 2C,D). Collectively, these data suggest that conditional deletion of *Tsn* or *Tsnax* from DA neurons does not affect the adiposity of the mice.

### 3.3. Effect of Conditional Deletion of Tsn or Tsnax from DA Neurons on Locomotor Response to Cocaine

Given that midbrain DA neurons are the principal targets of psychostimulants and that cocaine enhances dopaminergic signaling by inhibiting dopamine transporter (DAT) function [32], combined with our previous observation that global *Tsn* knockout (KO) mice exhibit normal acute but attenuated locomotor responses to repeated cocaine exposure, we investigated whether selective deletion of *Tsn* or *Tsnax* in DA neurons is required for normal locomotor responses to single or repeated cocaine administration.

**Figure 3:**
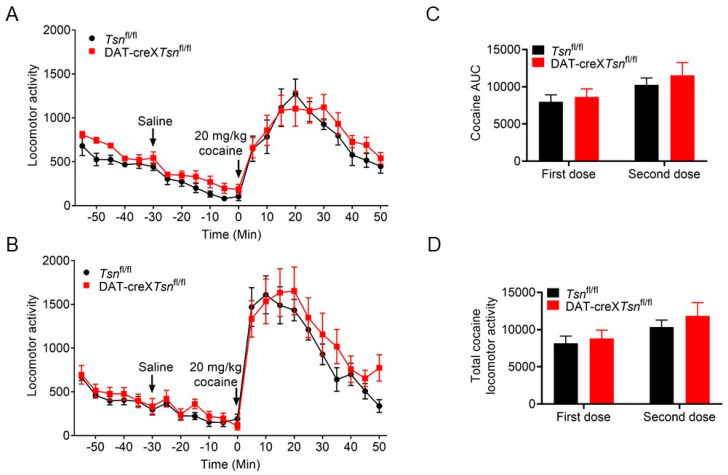
cKO of *Tsn* from DA neurons do not affect the cocaine-induced locomotor response. Cocaine-induced (20 mg/kg, i.p.) locomotor activity was monitored with *Tsn*^fl/fl^ and DAT-cre×*Tsn*^fl/fl^ mice the (**A**) first injection and the (**B**) second injection after two weeks. Locomotor activity was monitored every 5 min and arrowheads indicate the time of injections. (**C**) Area under the curve (AUC) and (**D**) total locomotor activity of the two injections of cocaine were calculated. n = 8/group. Values are presented as mean ± SEM. Statistical analysis was performed using two-way ANOVA with repeated measures, followed by Bonferroni’s post hoc test.

We first assessed the effects of conditional *Tsn* deletion in DA neurons on cocaine-induced locomotor activity. Mice from both the *Tsn*^fl/fl^ and DAT-cre×*Tsn*^fl/fl^ groups underwent cocaine treatment in two distinct sessions, with a two-week interval. No statistically significant differences in the locomotor activity were detected between the two genotypes during either injection session (Figure 3A,B). Furthermore, there were no significant differences in the area under the curve (AUC) or in total locomotor output during cocaine treatment between the groups (Figure 3C,D).

**Figure 4:**
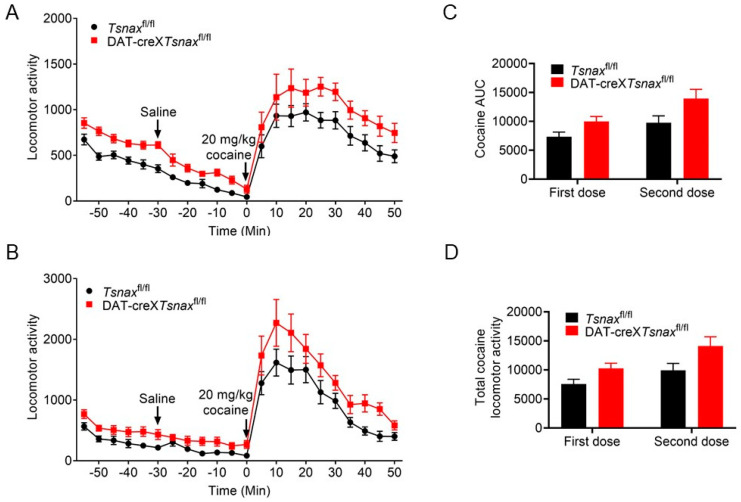
cKO of *Tsnax* from DA neurons do not affect cocaine-induced locomotor response. Cocaine-induced (20 mg/kg, i.p.) locomotor activity was monitored with *Tsnax*^fl/fl^ and DAT-cre×*Tsnax*^fl/fl^ mice the (**A**) first injection and the (**B**) second injection after two weeks. Locomotor activity was monitored every 5 min and arrowheads indicate the time of injections. (**C**) AUC and (**D**) total locomotor activity of the two injections of cocaine were calculated. n = 8/group. Values are presented as mean ± SEM. Statistical analysis was performed using two-way ANOVA with repeated measures, followed by Bonferroni’s post hoc test.

We also evaluated the impact of conditional *Tsnax* deletion in DA neurons on cocaine-induced locomotor responses. Although a trend towards increased locomotor activity was observed in DAT-cre×*Tsnax*^fl/fl^ mice compared to *Tsnax*^fl/fl^ controls following both single and repeated cocaine administration, statistical analysis revealed no significant differences (Figure 4A,B). Likewise, no statistically significant differences were detected in the AUC or in total locomotor activity during either the first or second cocaine exposure (Figure 4C,D). Collectively, these findings indicate that the conditional knockout of *Tsn* or *Tsnax* in DA neurons does not significantly affect locomotor responses to either acute or repeated cocaine administration.

### 3.4. Effect of Conditional Deletion of Tsn or Tsnax from DA Neurons on Locomotor Response to Amphetamine

Although both cocaine and amphetamine elicit locomotor stimulation by elevating dopaminergic tone, they do so via distinct molecular mechanisms. To further investigate the role of the TN/TX complex in DA neurons, we examined the effects of conditional deletion of *Tsn* or *Tsnax* on locomotor responses to both single and repeated amphetamine administration.

**Figure 5:**
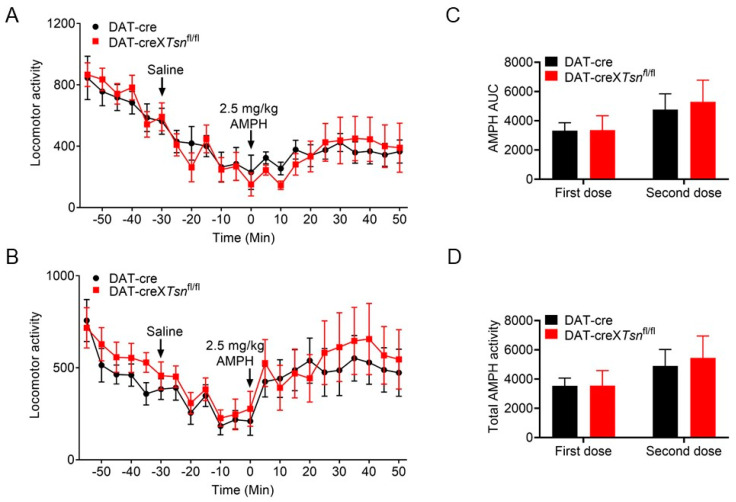
cKO of *Tsn* from DA neurons display normal does not alter amphetamine-induced locomotor response. Locomotor activity triggered by amphetamine (2.5 mg/kg, i.p.) was evaluated in DAT-Cre×*Tsn*^fl/fl^ and DAT-cre mice during (**A**) initial administration and (**B**) a second injection following a two-week interval. Activity was recorded at 5 min intervals with arrowheads indicating injection timepoints. (**C**) AUC and (**D**) total locomotor activity for both amphetamine challenges were quantified. n = 7–8 per group. Data represent mean ± SEM. Statistical analysis used two-way repeated measures ANOVA with Bonferroni post hoc testing.

Importantly, Chohan et al. [33] reported that hemizygosity for the DAT-Cre allele alone reduces sensitivity to amphetamine. Since our DAT-cre×*Tsn*^fl/fl^ or DAT-cre×*Tsnax*^fl/fl^ mice are hemizygous for DAT-Cre, we used DAT-Cre mice (without *Tsn* or *Tsnax* deletion) as controls. Consistent with our prior study [14], conditional deletion of *Tsn* in DA neurons resulted in locomotor responses to amphetamine that were indistinguishable from those of DAT-Cre controls (Figure 5A). To assess whether *Tsn* deletion affects locomotor sensitization, a second amphetamine injection was administered after a two-week interval. Locomotor activity remained comparable between DAT-cre×*Tsn*^fl/fl^ and control DAT-Cre mice (Figure 5B). Furthermore, no statistically significant differences were observed in the AUC or total locomotor output during amphetamine treatment across groups (Figure 5C,D).

**Figure 6:**
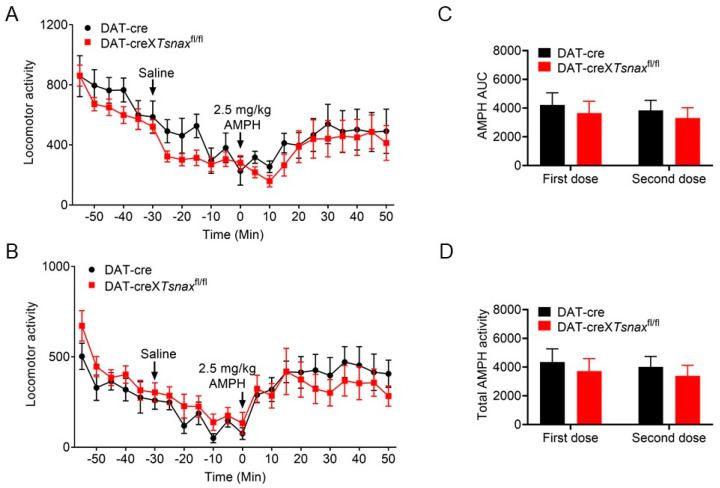
cKO of *Tsnax* from DA neurons display normal does not alter amphetamine-induced locomotor response. Locomotor activity triggered by amphetamine (2.5 mg/kg, i.p.) was evaluated in DAT-Cre×*Tsn*^fl/fl^ and DAT-cre mice during (**A**) initial administration and (**B**) a second injection following a two-week interval. Activity was recorded at 5 min intervals with arrowheads indicating injection timepoints. (**C**) AUC and (**D**) total locomotor activity for both amphetamine challenges were quantified. n = 8 per group. Data represent mean ± SEM. Statistical analysis used two-way repeated measures ANOVA with Bonferroni post hoc testing.

We next evaluated locomotor responses to amphetamine following conditional deletion of *Tsnax* in DA neurons. Consistent with the *Tsn* deletion phenotype, no significant differences emerged between DAT-cre×*Tsnax*^fl/fl^ and control DAT-Cre mice in either acute or repeated amphetamine-induced locomotion (Figure 6A–D). Taking them together, these findings indicate that the TN/TX complex in DA neurons is not required for modulating locomotor responses to either single or repeated amphetamine exposure.

### 3.5. Effect of Conditional Deletion of Tsn or Tsnax from DA Neurons on Amphetamine-Induced CPP

**Figure 7:**
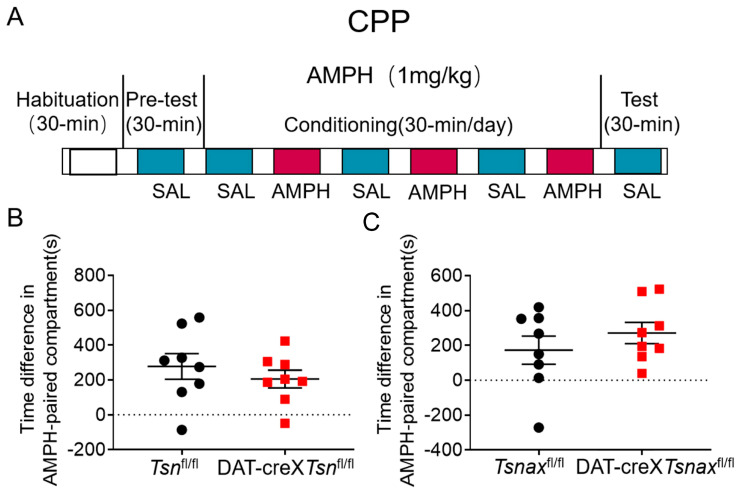
cKO of *Tsn* or *Tsnax* from DA neurons do not affect amphetamine-induced CPP. (**A**) CPP experimental scheme. (**B**) Time difference in AMPH-paired compartment between *Tsn*^fl/fl^ and DAT-cre×*Tsn*^fl/fl^ mice. (**C**) Time difference in AMPH-paired compartment between *Tsnax*^fl/fl^ and DAT-cre×*Tsnax*^fl/fl^ mice. n = 8/group. Values are shown as mean ± SEM, and statistical comparisons were made using Student’s *t*-test.

To investigate the role of the TN/TX complex in reward-associated memory, we evaluated whether conditional knockout of *Tsn* or *Tsnax* in DA neurons affects amphetamine-induced CPP. CPP (1 mg/kg amphetamine, i.p.) was assessed by using the protocol shown in the diagram (Figure 7A), and the results showed that there are no significant differences between *Tsn*^fl/fl^ and DAT-cre×*Tsn*^fl/fl^ mice or *Tsnax*^fl/fl^ and DAT-cre×*Tsnax*^fl/fl^ mice (Figure 7B,C), indicating that conditional knockout of *Tsn* or *Tsnax* from DA neurons does not impair the formation of AMPH-induced reward-associated memory.

## 4. Discussion

We have previously shown that TN and TX are concurrently expressed in midbrain DA neurons [14]. Given the pivotal role of these neurons in mediating behavioral responses to psychostimulants, we investigated the specific contributions of the TN/TX complex to neuronal function. Using conditional knockout strategies, we selectively ablated *Tsn* and *Tsnax* in DA neurons to delineate their roles. Surprisingly, despite the robust expression of TN/TX in DA neurons, conditional knockout of *Tsn* or *Tsnax* resulted in: (1) no alterations in locomotor responses to acute or repeated administration of cocaine or amphetamine; (2) no effect on amphetamine-induced CPP; and (3) no changes in adiposity. These findings suggest that the TN/TX complex may have yet uncharacterized functions in DA neurons. Interestingly, we observed asymmetric protein dependency: deletion of *Tsn* led to loss of TX expression, whereas *Tsnax* deletion did not affect TN levels. This provides a novel mechanistic insight into the distinct roles and interdependence of TN and TX.

### 4.1. Role of TN and TX in Cocaine or Amphetamine-Induced Behaviors

It is well established that both cocaine and amphetamine induce locomotor activation by enhancing dopaminergic signaling. Our previous studies showed that global *Tsn* KO mice exhibit normal locomotor responses to acute cocaine administration [24], with no significant changes in dopamine levels, metabolite ratios (DOPAC/DA, HVA/DA) in the nucleus accumbens (NAc), striatum, or prefrontal cortex, nor in the expression of dopamine transporter (DAT) or tyrosine hydroxylase (TH) in the NAc and striatum [14,24]. Given that increased adiposity in global *Tsn* KO mice confounds amphetamine pharmacokinetics, we utilized a conditional adult-onset global *Tsn* KO model that maintains normal adiposity to overcome this limitation. In that model, we previously demonstrated that TN is not required for amphetamine-induced locomotor activity [14]. Consistent with these findings, our current results show that conditional knockout of *Tsn* or *Tsnax* in DA neurons does not affect locomotor responses to acute psychostimulant exposure. Together, these data suggest that the TN/TX complex is not essential for mediating the acute effects of cocaine or amphetamine on dopamine signaling and associated locomotor behaviors.

Repeated exposure to psychostimulants such as amphetamine or cocaine induces behavioral sensitization in rodents, characterized by progressively enhanced locomotor and stereotypic behaviors [34]. This phenomenon is widely attributed to dopamine-mediated neuronal plasticity within VTA and NAc [35,36]. TN/TX complex has been implicated in the regulation of long-term synaptic plasticity via protein synthesis-dependent mechanisms [25], consistent with our previous findings that global deletion of *Tsn* attenuates cocaine sensitization [24]. In that study, we also observed that repeated cocaine administration upregulated *Tsnax* (TX) mRNA in D2-type medium spiny neurons (MSNs) in the NAc, and that this effect was partially mediated by RGS8, a known target of miRNAs elevated in *Tsn* KO mice. Crucially, the current study demonstrates that conditional deletion of Tsn or Tsnax in DA neurons does not alter locomotor sensitization to repeated cocaine/amphetamine (Figure 3, Figure 4, Figure 5 and Figure 6) or affect amphetamine-induced CPP (Figure 7). This spatial dissociation reveals two key principles: (1) TN/TX in DA neurons is not required for psychostimulant sensitization or reward memory; (2) The complex likely mediates plasticity in postsynaptic targets (e.g., striatal MSNs)—supported by its functional linkage to NAc miRNAs/RGS8 [24] and DA neuron-independent sensitization deficits observed in global KO. To test this model, future work will implement cell-type-specific Tsn KO in D1-/D2-MSNs, a logically compelled next step based on the exclusion map established in this study.

### 4.2. Role of TN and TX in Adiposity

DA neurons form the mesocorticolimbic reward pathway, originating in the ventral tegmental area (VTA) and projecting to limbic and cortical regions including the nucleus accumbens (NAc) and prefrontal cortex (PFC) [37,38]. While drug abuse (e.g., cocaine, amphetamine) robustly activates this circuitry, natural rewards—including food, sexual activity, and social interaction—also elicit dopamine release [39,40,41]. Importantly, dysregulated dopamine signaling has been implicated in maladaptive feeding behaviors. For instance, individuals carrying the DRD2 Taq1A1 allele exhibit reduced D2 receptor density, resulting in diminished reward signaling [42,43]. This deficiency may drive compensatory behaviors aimed at restoring dopaminergic tone, manifesting as substance abuse or excessive consumption of palatable, energy-dense foods that promote obesity and binge eating [44].

Our previous work demonstrated that the global *Tsn* KO mice exhibit increased adiposity [45], whereas hepatocyte- or adipocyte-specific knockout of *Tsn* or *Tsnax* does not replicate this phenotype [23]. To determine whether dopaminergic neurons mediate the adiposity phenotype, we assessed body fat composition in mice with *Tsn* or *Tsnax* selectively deleted in DA neurons. Notably, conditional knockout of either gene within this neuronal population failed to alter adiposity. These findings suggest that TN/TX regulates adiposity via mechanisms outside the dopaminergic system, shifting attention toward peripheral or extra-mesolimbic circuits.

### 4.3. Functional Independence of TN and TX

The TN/TX complex forms a hetero-octamer consisting of two TN homodimers and two TN/TX heterodimers [46,47]. While TN is capable of self-assembling into functional homo-octamers critical for DNA binding and repair, TX lacks intrinsic homomeric assembly capacity. Notably, our study uncovers a key asymmetry in complex stability: TX protein is nearly undetectable in *Tsn* knockout mice despite unaltered mRNA expression [14], suggesting that TX relies on interaction with TN for post-translational stability—likely through protection from proteasomal degradation. In contrast, *Tsnax* deletion does not impact TN protein levels, establishing that TN stability is independent of TX. This unidirectional dependency underscores TN’s role as a structural scaffold essential for TX function.

In addition to its RNase activity within the TN/TX complex, TX also performs TN-independent functions, most notably by binding and facilitating ATM autophosphorylation in response to DNA damage [48]. This duality—TN-dependent stabilization versus TN-independent signaling—raises a mechanistic paradox: how does TX transition between its complex-associated and independent functional states? Future studies will be needed to elucidate the structural mechanisms governing TX dissociation from the complex.

## 5. Conclusions

This study employed DA neuron-specific conditional knockouts of *Tsn* and *Tsnax* to examine the cell-autonomous functions of the TN/TX complex in metabolic and behavioral regulation. Despite robust TN/TX expression in midbrain DA neurons, their deletion did not alter psychostimulant-induced locomotion, amphetamine-conditioned place preference, or adiposity, suggesting that conditional deletion of TN/TX in DA neurons reveals no significant impact on psychostimulant behaviors or adiposity.

## Data Availability

The original contributions presented in this study are included in the article. Further inquiries can be directed to the corresponding author.
